# The Clinical Value of Huangqi Injection in the Treatment of Leucopenia: A Meta-Analysis of Clinical Controlled Trials

**DOI:** 10.1371/journal.pone.0083123

**Published:** 2013-12-12

**Authors:** Changsong Zhang, Changtai Zhu, Yang Ling, Xifa Zhou, Chunlei Dong, Judong Luo, Yongping Liu

**Affiliations:** 1 Department of Oncology, Changzhou Tumor Hospital Soochow University, Changzhou, China; 2 Department of Laboratory Medicine, Changzhou Tumor Hospital Soochow University, Changzhou, China; 3 Department of Radiation Oncology, Changzhou Tumor Hospital Soochow University, Changzhou, China; Cardiff University, United Kingdom

## Abstract

**Background:**

Huangqi injection is derived from Astragalus membranaceus root. In China, recent reports of Huangqi injection for the treatment of leucopenia have emerged. However, a systematic review of these reports has not been performed. Thus, we conducted a meta-analysis of clinical controlled trials to assess the clinical value of Huangqi injection in the treatment of leucopenia.

**Methods:**

We searched the Chinese Biomedical Literature Database (CBM), Wanfang Database, China National Knowledge Infrastructure (CNKI), Chinese Scientific Journals Full-text Database (VIP), as well as PubMed and EMBASE to collect the data about trials of Huangqi injection for treating leucopenia. A meta-analysis was performed using RevMan 5.2 software.

**Results:**

A total of 13 studies involving 841 patients were included in this study. The overall study quality was lower according to the Jadad scale. The meta-analysis showed that experimentally treated patients experienced greater therapeutic efficacy and lower white blood cell counts than control groups treated with Western medicine (*P* < 0.05). No publication bias was evident, according to Egger’s test.

**Conclusions:**

The validity of this meta-analysis was limited by the overall poor quality of the included studies. Huangqi injection may have potential clinical value in the treatment of leucopenia, but confirmation with rigorously well-designed multi-center trials is needed.

## Introduction

Leucopenia is defined by a lower-than-normal peripheral white blood cell (WBC) count. Leucopenia commonly arises from cancer chemotherapy or radiotherapy, viral infection, drug-induced reactions, and certain immune diseases [1-7]. Recent studies suggest that a single-nucleotide polymorphism may also cause leucopenia [8]. At present, leucogen, shark glycol, vitamin B4, and inosine have been used to treat leucopenia. However, these treatments fail in some cases, and novel approaches for treating leucopenia are needed. Recently, in China, Huangqi injection for the treatment of leucopenia has been reported in many clinical trials. These individual studies suggest that Huangqi injection may be useful for the treatment of leucopenia, but a systematic review has not been performed. Therefore, we conducted a meta-analysis of clinical controlled trials to assess the therapeutic value of Huangqi injection for the treatment of leucopenia.

## Materials and Methods

### Inclusion criteria

The clinical trials were clinically controlled studies and experimental groups were treated with Huangqi injection. Controls in the studies were treated with Western medicine as described in Table 1. Outcome measures were effectiveness rates and WBC counts. WBC measurements were performed by an independent laboratory and measured in SI units (10^9^/L). When total WBCs were greater than 4.0×10^9^/L or elevated more than 1.0×10^9^/L in the peripheral blood arising from a drug intervention, treatments were considered to be effective. Before treatment, the baseline peripheral WBCs were comparable between the experimental group (Huangqi injection) and the control group (Western medicine) (*P*>0.05). In this study, we restricted no clinical trials based on patient gender, race, or language of the publication.

**Table 1:** Characteristics of the individual trials included in this study.

**Author [Reference]**	**Published year**	**Cases E/C**	**Age (years) Range, mean**	**Sex Male/female**	**Etiopathogenesis**
Zhang MJ[15]	1999	35/32	E: 18-50, 33 C: 20-56,37	E: 7/28 C: 19/23	Radiotherapy, chemotherapy, immunodiseases
Dai Y[17]	1999	68/27	25-76,55.6	59/36	Radiotherapy, chemotherapy
Feng CL[10]	2000	32/28	E: 40-79 C: 42-79	E: 14/18 C: 13/15	Infection
Zhang YS[14]	2000	58/54	E: 16-52,36 C: 15-56,34.6	E: 30/28 C: 32/22	Radiotherapy, chemotherapy, drug use, etc
Gao P[16]	2000	30/25	E: 15-56,32 C: 18-55,35	E: 10/20 C: 9/46	Radiotherapy, chemotherapy, viral infection, immunodiseases, etc
Qu W[21]	2000	32/28	E: 16-63,38.2 C: 15-56,34.6	E: 14/18 C: 12/16	Graves disease with tapazole
Yang DS[11]	2001	25/25	18-55,33.5	21/29	Radiotherapy, chemotherapy, drug use
Mo WG[18]	2005	30/30	E: 0-12,2.44 C: 0-11,2.99	E: 19/11 C: 17/13	Viral infection
Tang JC[13]	2007	23/22	13-64,50	30/15	Nasopharyngeal carcinoma patient with radiotherapy
Wang HJ[20]	2007	40/39	16-68,31.5	6/73	Systemic lupus erythematosus
Xiao AQ[9]	2007	26/25	33-73	E: 14/12 C: 15/10	Ticlopidine use
Mo WX[19]	2009	20/20	E:16-54,28 C:18-52,26	E: 8/12 C: 9/11	Schizophrenia with clozapine
Qin HZ[12]	2011	35/32	E: 19-51,32 C: 22-55,36	E: 9/26 C: 10/22	Radiotherapy, chemotherapy, viral infection, immunodiseases, etc

E: experimental group, C: control group.

### Exclusion criteria

Duplicated literature, reviews, non-clinical studies, case observations, and non-injection formulae literature were excluded in this study.

### Research strategy and data extraction

**Figure 1:**
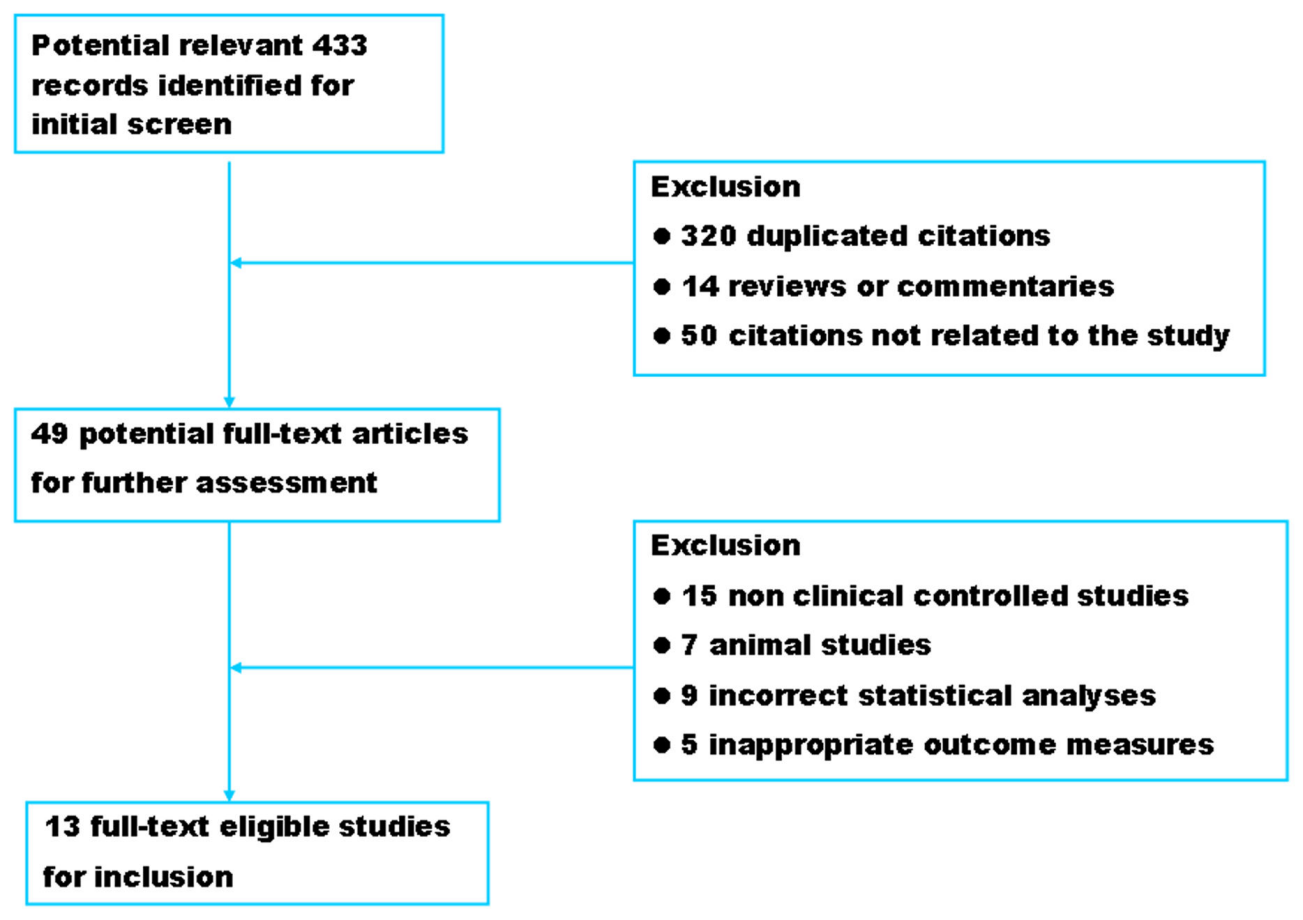
Flow diagram of literature retrieval.

“Huangqi” or “huang qi” or “astragalus” or “astragali” or “astragalus miltiorrhiza” or “Chinese traditional medicine herb” AND “leucopenia” or “leucocytopenia” or “leucopenia” or “aleucocytosis” or “hypolekocytosis” or “hypoleucocytosis” or “hypoleukemia” or “hypoleukia” or “hypoleukocytosis leucocytopenia” or “leucopenia” or “oligoleukocythemia” or “oligoleukocytosis” or “white blood count” were selected as the free-text terms or MeSH terms. The Chinese Biomedical Literature Database (CBM), Wanfang Database, China National Knowledge Infrastructure (CNKI), Chinese Scientific Journals Full-text Database (VIP), PubMed, and EMBASE were searched. Data extraction and quality assessment were independently performed by two researchers [CTZ and YL] and any discrepancies were resolved by consensus or in consultation with a third reviewer [CSZ]. The lack of information in any study was supplemented by contact with the authors in charge of the clinical trials. The database retrieval process is shown in Figure 1.

### Statistical analysis

Statistical analysis was performed using Cochrane RevMan 5.2 and STATA 11.0 software. Categorical variables were compared using odds ratios (OR), and continuous variables were compared using standard mean differences (SMD). A 95% confidence interval (CI) was calculated and the Chi-square test was used for heterogeneity of inclusion trials. Data of heterogeneity were studied using a random effects model; otherwise a fixed effects model was used. A funnel plot and Egger’s test were employed to judge potential publication bias.

## Results

### Characteristics of included studies

Thirteen articles [9-21] involving 841 subjects (experimental groups: 454 cases; the control groups: 387 cases) were included in this study. Causes of leucopenia were mainly viral infection, cancer chemotherapy, drug-induced reactions, and immune disorders. The general characteristics of the study are shown in Table 1, and interventions, treatments, and outcomes are depicted in Table 2. 

**Table 2:** The intervention measures of the individual studies included in this study.

**Author**	**The regimens of intervention**		**Treatment**
**[reference]**	**Experimental group**	**Control group**	**Time**
Zhang MJ[15]	Huangqi injection (40 ml) were respectively added to a 5% glucose solution (250 ml), intravenously, qd	Oral leucogen, and batyl alcohol; energy mixture, intravenously	30 d
Dai Y[17]	Huangqi injection (40 ml) were respectively added to a 5% glucose solution (250 ml), intravenously, qd	Oral batyl alcohol and leucogen	7-10 d
Zhang YS[14]	Huangqi injection (40 ml) were respectively added to a 5% glucose solution (500 ml), intravenously, qd	Oral inosine, leucogen and batyl alcohol	14 d
Gao P[16]	Huangqi injection (40 ml) were respectively added to a 5% glucose solution (250 ml), intravenously, qd	Oral batyl alcohol, tid	30 d
Tang JC[13]	Huangqi injection (40 ml) were respectively added to a 5% solution (250 ml), intravenously, qd	Oral inosine	49 d
Mo WX[19]	Huangqi injection (20 ml) were respectively added to a 5% solution (250 ml), intravenously, qd	Oral leucogen and vitamin B4	45 d
Qin HZ[12]	Huangqi injection (40 ml) were respectively added to a 5% solution (250 ml), intravenously, qd	Oral leucogen and batyl alcohol, energy mixture, intravenously	30 d
Feng CL[10]^#^	Huangqi injection (20-40 ml) were respectively added to a 5‑10% glucose solution (250 ml), intravenously, qd	Anti-infecion and symptomatic treatment	15–21 d
Qu W[21]^#^	Huangqi injection (40 ml) were respectively added to a 5% solution (250 ml), intravenously, qd	Oral tapazole	28 d
Yang DS[11]^#^	Huangqi injection (40 ml) were respectively added to a 5% solution (250 ml), intravenously, qd	Oral vitamin B4 and leucogen	30 d
Mo WG[18]^#^	Huangqi injection (5-10ml) were respectively added to a 5% solution (250 ml), intravenously, qd	Conventional antiviral and symptomatic treatment	7 d
Wang HJ[20]^#^	Huangqi injection (40 ml) were respectively added to a 5% solution (250 ml), intravenously, qd	Oral prednisone and leflunomide	2–4 w
Xiao AQ[9]^#^	Huangqi injection (40 ml) were respectively added to a 5% solution (250 ml), intravenously, qd	Oral leucogen and batyl alcohol	30 d

^#^: In the experimental group, interventional regimens included drugs of the control group other than Huangqi injection.

### The quality assessment

The Jadad scale was scored by randomization, randomization methodology, double-blinding, withdrawals/dropouts, and allocation concealment [22-24]. The Jadad scores ranged from 0 to 2 (Table 3), suggesting that the overall quality of the literature was lower.

**Table 3:** Quality of clinical trial reports using the Jadad assessment scale.

**Author[reference]**	**Randomization**	**Randomization methodology description**	**Double-blinding**	**Withdrawals/dropouts**	**Allocation concealment**	**Scores**
Zhang MJ[15]	No	No	No	No	No	0
Dai Y[17]	Yes	No	No	No	No	1
Feng CL[10]	No	No	No	No	No	0
Zhang YS[14]	Yes	No	No	No	No	1
Gao P[16]	Yes	No	No	No	No	1
Qu W[21]	Yes	No	No	No	No	1
Yang DS[11]	Yes	Yes	No	No	No	2
Mo WG[18]	No	No	No	No	No	0
Tang JC[13]	Yes	No	No	No	No	1
Wang HJ[20]	Yes	No	No	No	No	1
Xiao AQ[9]	Yes	No	No	No	No	1
Mo WX[19]	Yes	No	No	No	No	1
Qin HZ[12]	Yes	No	No	No	No	1

### Meta-analyses of the effectiveness of Huangqi injection

**Figure 2:**
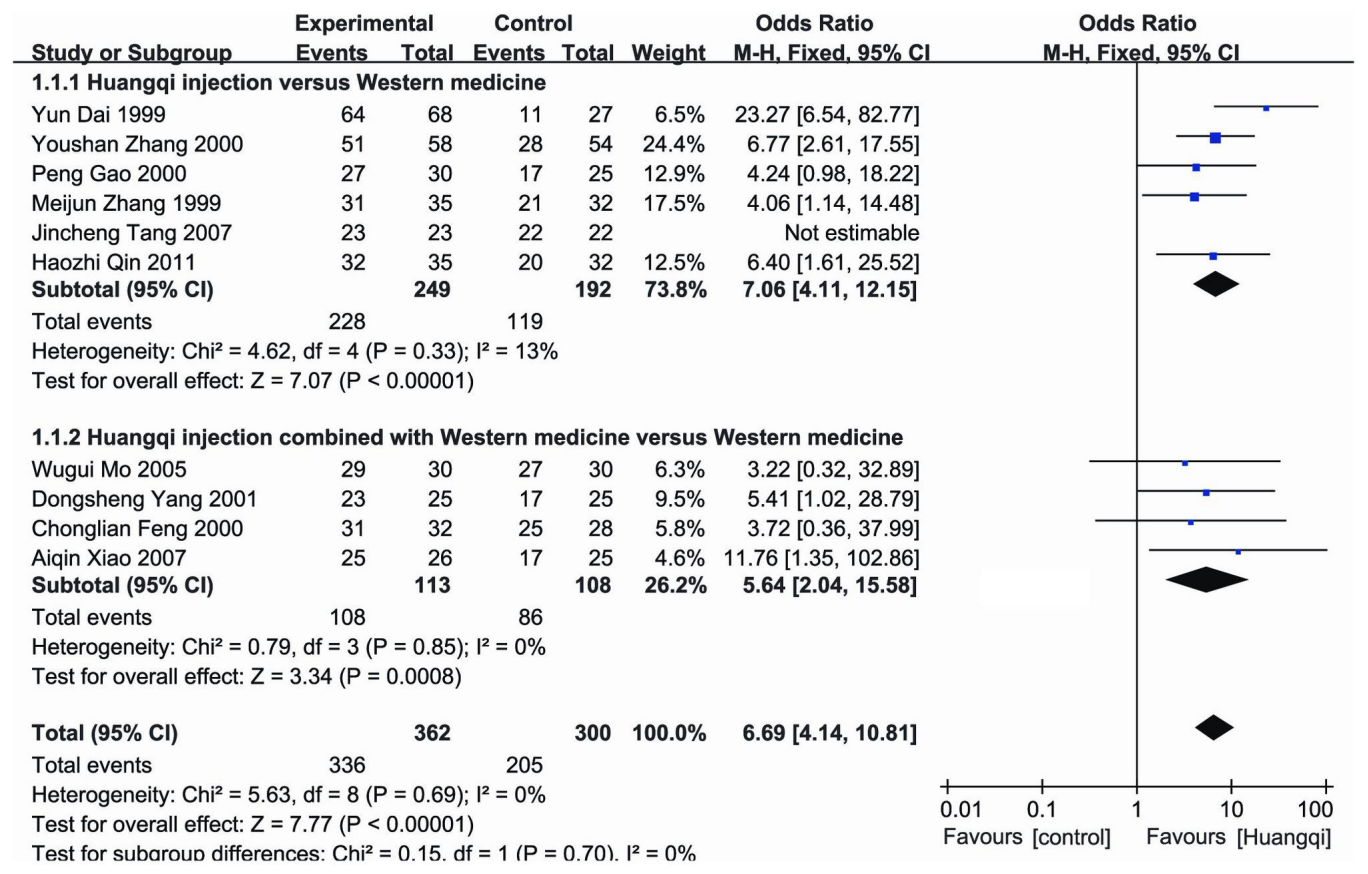
Effectiveness of Huangqi injection for the treatment of leucopenia. Events: the numbers of the effective cases.

**Figure 3:**
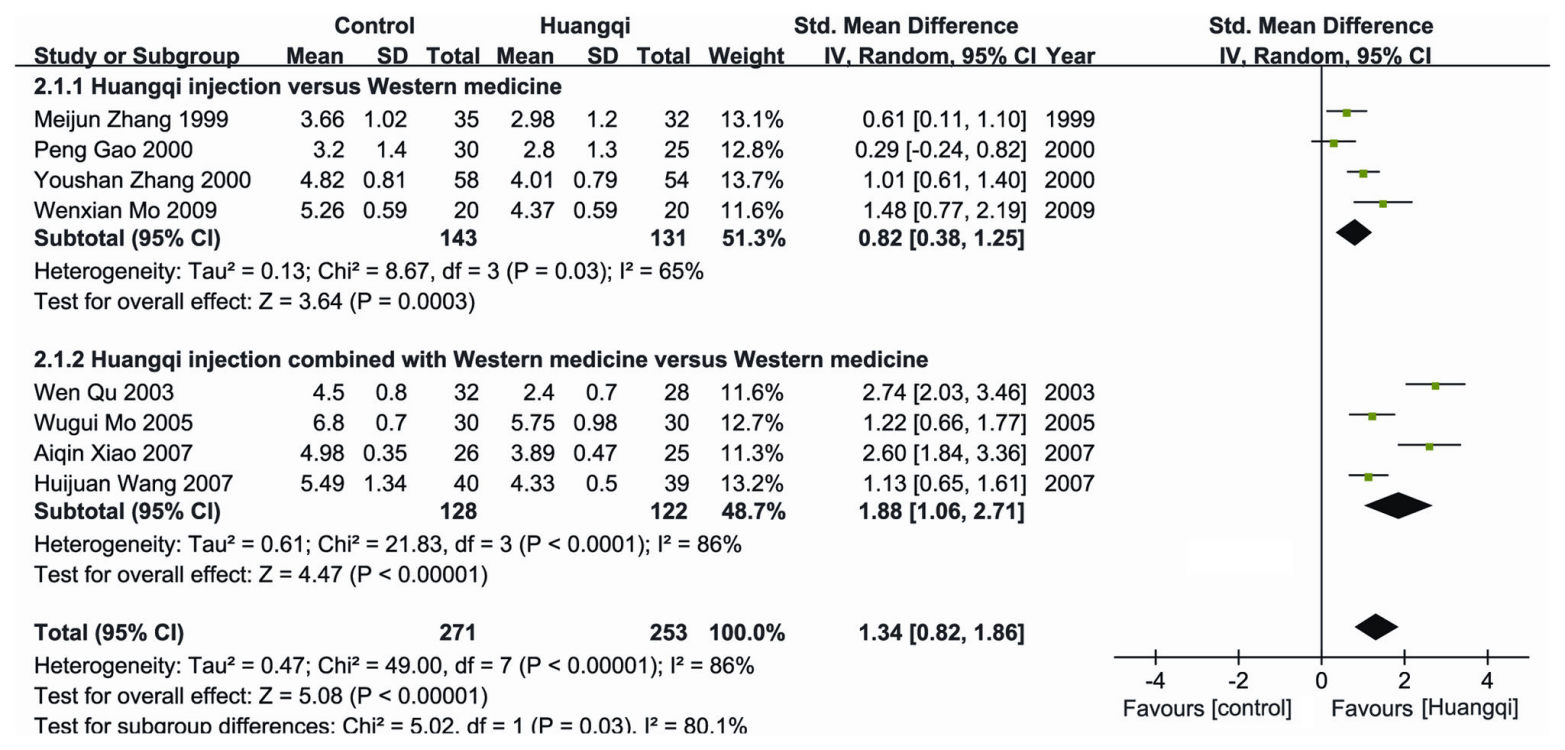
Effect of Huangqi injection on white blood cell.

Ten studies [9-18] described the effectiveness of Huangqi (Figure 2). Meta-analysis revealed an overall effectiveness rate in the experimental group that was higher than that in the control group [OR = 6.69, 95% CI (4.14, 10.81), *P* < 0.05; fixed effects model] (Figure 2). The combined estimates of WBCs in the experimental group was also higher than that in the control group [SMD=1.94, 95% CI (1.19-2.69), *P* < 0.05; random effects model] (Figure 3).

### Subgroup analyses of Huangqi injection versus Western medicine

The pool effectiveness rate in Huangqi injection treatment group was higher than that in the Western medicine treatment group [OR = 7.06, 95% CI (4.11, 12.15), *P* < 0.05; fixed effects model] (Figure 2). WBC counts in the Huangqi injection treatment group were higher than those in the control group [SMD=0.82, 95% CI (0.38, 1.25), *P* < 0.05; random effects model] (Figure 3). 

### Subgroup analyses of Huangqi injection combined with Western medicine versus Western medicine

There was significant difference in the pool effectiveness rate between the experimental group with Huangqi injection combined with Western medicine and the control group with Western medicine [OR = 5.64, 95% CI (2.04, 15.58), *P* < 0.05; fixed effects model] (Figure 2). WBCs in the experimental group were significantly higher than those in the control group [SMD=1.88, 95% CI (1.06, 2.71), *P*<0.05; random effects model] (Figure 3). 

### Side effects

In this study, five reports [9,12,13,16,19] confirmed that no side effects in clinical trials were observed. The remaining reports did not mention adverse effects. Thus, we could not evaluate adverse effects due to this deficit.

### Publication bias

**Figure 4:**
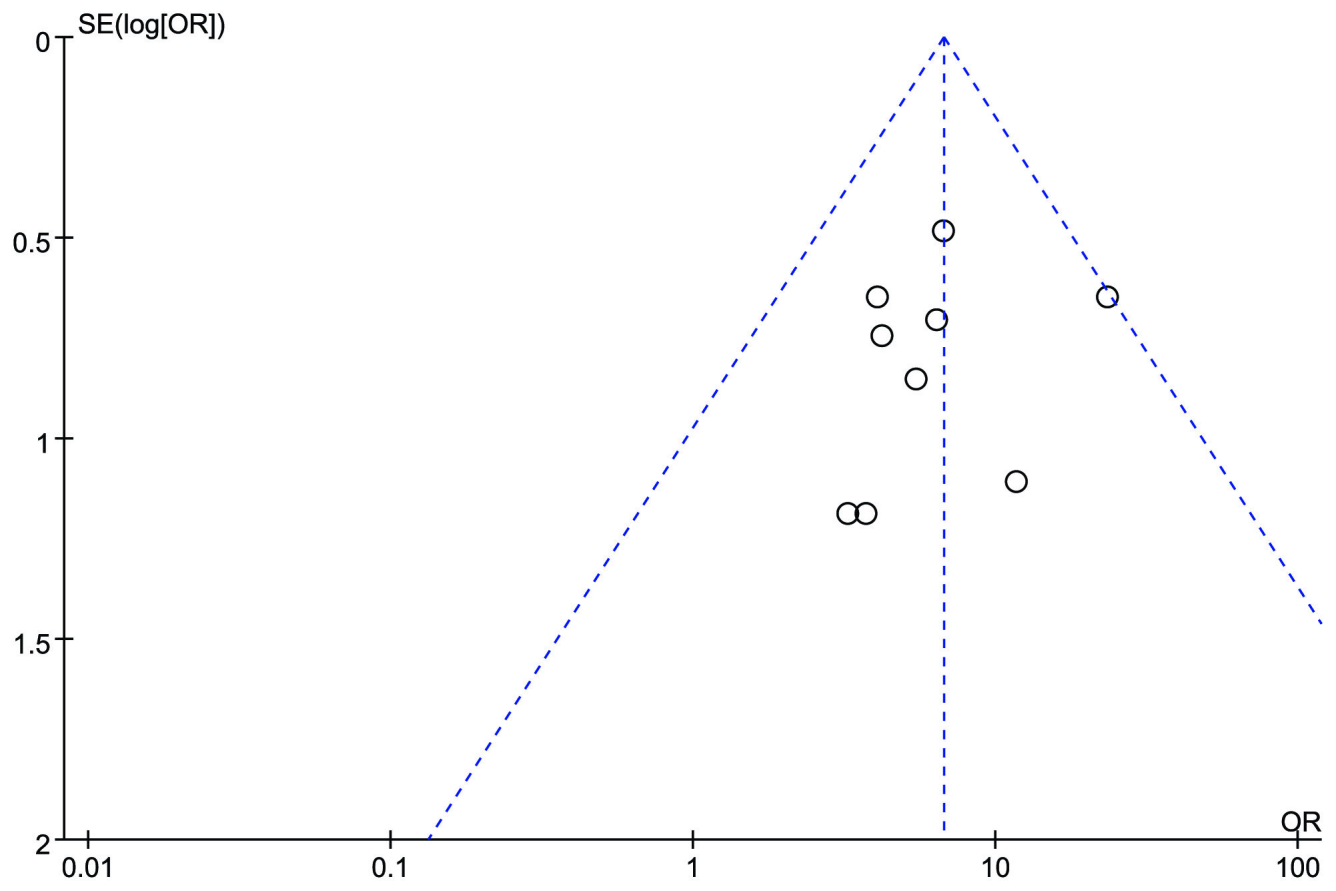
Funnel plots based on data of overall effectiveness.

There was no publication bias according to the results of Egger’s test performed by STATA 11.0 (*P* = 0.264). The funnel plot drawn by Cochrane Revman 5.2 was basically symmetric (Figure 4).

## Discussion

Recently, in China, apart from treating leucopenia, Huangqi injection has been widely used to treat chronic hepatitis [25-27], cirrhosis [28], chronic heart failure [29,30], chronic nephritis [31-33], and diabetic nephropathy [34,35]. 

Huangqi injection is derived from the *Radix Astragali Mongolici* root using ethanol. Modern pharmacological studies demonstrated that astragalus flavonoids, an effective component of Huangqi, can eliminate radiation toxicity and increase granulocyte colony-stimulating factor to promote stem cell proliferation [36]. In addition, Huangqi can regulate humoral immunity, inhibit proliferation of tumor cells and reduce chemotherapeutic toxicity [37-41]. 

According to this meta-analysis, Huangqi injection was more efficacious than the Western medicine control group. Subgroup analyses revealed that the overall effectiveness rates in the experimental group receiving Huangqi injection alone or combined with Western medicine was higher compared with Western medicine alone. 

We noted that five studies suggested that clinical trial side effects were not observed. Whereas, according to a review of 83 studies [42] regarding adverse reactions in response to Huangqi injection (1995–2008) by the Evidence Based Medicine Centre, Tianjin University of Traditional Chinese Medicine, China, data suggest that only moderate reactions were observed. Most of these were dermal allergenic reactions; no deaths were reported. These findings suggest that Huangqi may be safe and may have a potential clinical value in the treatment and prevention of leucopenia. Considering reports that Huangqi injection could enhance immunity and inhibit tumors, use of Huangqi to treat cancer patients during radiotherapy and chemotherapy may be possible. Interestingly, the previous Cochrane systematic review [43] reported that the proportion of patients with leucopenia was significantly lower in the groups treated with Huangqi injection in addition to chemotherapy and the pooled estimate of absolute rate reduction was 36% (95% CI: 21% to 51%), suggesting that Huangqi injection may be used as a preventative treatment for leucopenia during chemotherapy. However, in this present study, we didn’t conduct a meta-analysis on the preventative treatment.

Although no publication bias was confirmed according to the results of Egger’s test, the meta-analysis did have some limitations. These may decrease the validity of evidence-based medicine within this meta-analysis. The main limitations were poor quality of included studies and in our meta-analysis, no study accounted for withdrawals/dropouts and only one provided a description of randomization methodology. Neither double-blind nor concealed allocation studies were mentioned in the included literature. Moreover, available data about follow-up regarding long-term efficacy and safety were insufficient. Based on these disadvantages, a treatment effect of this size seems quite unlikely. It must be emphasized that such trials should be randomized and ideally placebo controlled.

Fortunately, in the included studies, WBC counts were performed by independent laboratory personnel and the staff was blinded to the treatment drug group/patient group. These data suggest that the outcome evaluation was reliable.

Presently, most clinical trials published in Chinese medical journals were of poor quality with respect to methodological design according to the Jadad scale [41,44]. Future trials to evaluate Huangqi should incorporate clinical trial registration and adoption of the international reporting criteria such as CONSORT, STROBE and PRISMA. These procedures would drastically elevate the validity of the clinical trials. 

## Conclusions

The validity of this meta-analysis was limited by the overall poor quality of the included studies. Huangqi injection may have potential clinical value in the treatment of leucopenia, but further studies are warranted, especially rigorous and well-designed multi-center trials.

## Supporting Information

Checklist S1 **PRISMA Checklist.**
(DOC)
